# Public stigma toward fatigue—do social characteristics of affected persons matter? Results from the SOMA.SOC study

**DOI:** 10.3389/fpsyg.2023.1213721

**Published:** 2023-08-11

**Authors:** Olaf von dem Knesebeck, Rieke Barbek

**Affiliations:** Institute of Medical Sociology, University Medical Center Hamburg-Eppendorf, Hamburg, Germany

**Keywords:** fatigue, public stigma, stereotypes, anger, intersectionality, social inequalities, SOMA.SOC

## Abstract

**Objectives:**

Although public stigma imposes a great burden on those affected with manifold negative consequence there is not much known about public stigma related to fatigue. Therefore, this study addresses the following research questions: (1) What is the magnitude of public stigma toward individuals with fatigue? (2) Are there differences in public stigma depending on the social characteristics of the affected person (sex, occupation, and migration)?

**Methods:**

Analyses are based on a national telephone survey in Germany (*N* = 1,209). A vignette describing a person with symptoms of fatigue was presented to the respondents. Vignettes were varied according to sex, occupational status, and migration status. Stigma toward the person presented in the vignette was measured by stereotypes and negative emotional reactions (anger).

**Results:**

Of the stereotypes under study, “hypersensitive” was most frequently endorsed by the respondents (35.7%), followed by “weak will” (27.2%). About 15–20% of the respondents agreed that they react with anger, annoyance or incomprehension. There were considerable differences in fatigue stigma according to the social characteristics of the affected person. In two social groups public stigma was particularly pronounced: (1) male persons with a low occupational status and a migration background; (2) female persons with a high occupational status and without a migration status. In contrast, women with a low occupational status and a migration background were less stigmatized.

**Conclusion:**

Individuals suffering from fatigue symptoms are confronted with stereotypes and negative emotional reactions by the public. Magnitude of public stigma considerably varies according to social characteristics of the afflicted person. Future studies should consider applying an intersectional approach to identify groups that are at risk of multiple stigma.

## Introduction

According to Link and Phelan (2001), stigma is defined as a process in which labeling, stereotyping, status loss and discrimination co-occur in a situation where power is exercised. Public stigma is usually assessed by different beliefs endorsed by the general population, such as the ascription of attributes (stereotypes), emotional response upon meeting an afflicted person (e.g., anger or fear) or the desire to socially distance oneself (Knesebeck et al., 2015). Public stigma imposes a great burden on those affected with negative consequences for their health (Alvarez-Galvez and Salvador-Carulla, 2013; Schomerus et al., 2015) and help-seeking behavior (Griffiths et al., 2011; Clement et al., 2015). Moreover, stigma can be a barrier that impedes diagnosis of somatoform and related disorders (Murray et al., 2016).

Many somatic symptoms cannot exclusively ascribed to somatic diseases (e.g., vascular or inflammatory disease) or mental disorders (e.g., depressive or anxiety disorders) (Löwe et al., 2022). In this regard, a dualistic view classifying symptoms as either somatic or psychological is often inappropriate. With reference to the description of bodily distress disorder in the International Classification of Diseases, 11th edition (World Health Organization, 2023), persistent somatic symptoms are defined as being present on most days for at least several months. Fatigue is a persistent somatic symptom with an estimated prevalence of about 30% among adults in Germany (Kocalevent et al., 2011). It can be expected that this prevalence has increased in the last years as fatigue is a frequent symptom of long COVID (Global Burden of Disease Long COVID Collaborators, 2022). It has been reported that persons afflicted by fatigue symptoms may carry a burden of stigma because their symptoms are poorly understood and often unrecognized or unacknowledged by health professionals and the public (Green et al., 1999; Jason et al., 2004). Most empirical studies examining stigma related to fatigue primarily focus on perceived or anticipated stigma, and not on public stigma. In these studies, patients are asked whether they experienced or anticipate to be stigmatized because of their symptoms (e.g., Grover et al., 2021; Froehlich et al., 2022). In this regard, a recent review (Ko et al., 2022) explored the differences between patients with a chronic fatigue syndrome (CFS) and patients with comparable explained conditions concerning perceived and experienced stigma. Results based on the two studies (Looper and Kirmayer, 2004; Baken et al., 2018) included in the review showed that patients with CFS experienced higher levels of stigma. One study on public stigma toward people affected by somatic symptom disorder (SSD, Knesebeck et al., 2018) used vignettes that differed regarding main type of symptom (pain vs. fatigue) and existence of an earlier disease (yes vs. no). Results indicated public stigma (stereotypes, negative emotional reactions, and desire for social distance) toward people affected by SSD in Germany. Magnitude of SSD stigma was similar, irrespective of main type of symptom and existence of an earlier disease. Altogether, there is a lack of research on public stigmatizing beliefs toward individuals affected by fatigue.

Many studies look at health-related stigma without considering other (social) conditions that also may be associated with stigmatization (e.g., poverty, low socio-economic status, ethnic minority). The circumstance that individuals potentially belong to more than one stigmatized group is conceptualized as “layered stigma” (Henkel et al., 2008), “multiple stigma” (Makowski et al., 2019), or “intersectional stigma” (Turan et al., 2019). These concepts suggest that a convergence of multiple stigmatized identities within a person or group will result in joint or cumulative effects. Empirical studies examining this hypothesis yielded mixed findings (Grollman, 2015; Knesebeck et al., 2017; Makowski et al., 2019; Turan et al., 2019). Women, ethnic minorities and people with a low socio-economic status (SES) are often mentioned groups potentially affected by layered stigma (Gary, 2005; Henkel et al., 2008; Pecosolido and Martin, 2015; Turan et al., 2019). However, to our knowledge there is no study that empirically analyses whether individuals belonging to these groups and afflicted by fatigue are confronted with multiple stigma. Therefore, we address the following research questions: (1) What is the magnitude of public stigma in terms of stereotypes and anger toward individuals with fatigue? (2) Are there differences in public stigma depending on the social characteristics of the affected person (sex, occupation, and migration)?

## Materials and methods

### Study design and sample

Analyses are based on cross-sectional data collected between March and May 2022 via a telephone survey (computer assisted telephone interview) of the adult population (age ≥ 18 years) living in Germany. About 70% of the sample was drawn from all registered private telephone numbers at random, additional computer-generated numbers allowed for inclusion of ex-directory households (landline numbers). The other 30% of the sample consisted of randomly generated mobile phone numbers (Random Digit Dialling). For a random selection of participants in the households, the Kish-Selection Grid was applied (Kish, 1949). Oral informed consent was given in the beginning of the interview. In total, *N* = 2,413 individuals participated in the survey, reflecting a response rate of 45%. To gain a representative sample of the adult population living in Germany, the data set was weighted. Comparison of sociodemographic characteristics of the sample with official statistics indicates that distribution of age, gender, and education is similar to the general adult population in Germany.

The survey is part of a project on social inequalities in aggravating factors of persistent somatic symptoms (SOMA.SOC, Knesebeck et al., 2023) which is embedded in the Research Unit 5211 “Persistent SOMAtic Symptoms ACROSS Diseases: From Risk Factors to Modification (SOMACROSS)” (Löwe et al., 2022). The study design was approved by the Ethics Commission of the Hamburg Medical Chamber (No. 2020-10194-BO-ff).

### Vignettes

At the beginning of the interviews, a vignette describing a person with symptoms of fatigue or irritable bowel syndrome (IBS) was presented to the respondents in the survey. We chose these two specific conditions as they appear relatively frequently in the German population and other projects of the research unit also focussed on fatigue and IBS. Vignettes were developed with the input of clinicians (colleagues of the SOMACROSS research unit, i.e., specialists from psychosomatic, general and internal medicine) considering typical symptoms described in the International Classification of Diseases (DIMDI, 2019). One of the two vignettes (fatigue or IBS) was randomly assigned to half of the sample, respectively. As the following analyses will focus on fatigue, half of the total sample (*n* = 1,209) will be used. In terms of the fatigue vignette, different symptoms like exhaustion, weakness, and cognitive limitations were described (DIMDI, 2019; AWMF, 2022, please see Appendix). To examine differences in public stigma, vignettes were varied according to sex (male/female), occupational status (high/low), and migration status (yes/no). Thus, eight different fatigue vignettes were used (please see Table 1) that each were randomly assigned to about 150 respondents (i.e., about 12.5% of the analysed sample). A sample size of *n* = 150 per vignette allowed for the detection of small to medium differences (statistical power 80%, Type-I error = 0.05). In terms of migration status, the person in the vignette had a Turkish name and it is said that the person came from Turkey to Germany 10 years ago. Turkey was chosen as country of origin because about 2.9 million inhabitants in Germany with a Turkish background are forming the largest migrant group (Schürer, 2018). In the high occupational status vignette, it is said that the respective person is a lawyer and in the low status vignette the person is a cleaner. The vignettes were audio-recorded with a trained speaker with a clear voice. In order to increase reliability and to neutralize possible interviewer-associated effects, this file was presented to the respondents directly from the computer via telephone line. We used unlabelled vignettes, i.e., the respondents were not informed that the person in the vignette had fatigue.

**Table 1:** Eight variations of the fatigue vignette presented in the survey.

	Combination of social factors
1	Male, migration history, lawyer
2	Male, no migration history, lawyer
3	Male, migration history, cleaner
4	Male, no migration history, cleaner
5	Female, migration history, lawyer
6	Female, no migration history, lawyer
7	Female, migration history, cleaner
8	Female, no migration history, cleaner

### Indicators

To assess stigma toward the person presented in the vignette, two components of the stigma process [1] were considered: stereotypes and negative emotional reactions (anger). In terms of stereotypes, respondents were asked to indicate to what extent they agree or disagree with the following statements (McLoed et al., 2007; Angermeyer et al., 2013; Knesebeck et al., 2018) on a four-point Likert scale: (1) “People with symptoms like Mrs./Mr. E. are hypersensitive.” (2) “Mrs./Mr. E. does not have a real disease.” (3) “A possible cause for the symptoms of Mrs./Mr. E. is a weak will.” Based on a study of Angermeyer and Matschinger (2003), three items (“I react angrily.,” “I feel annoyed by this person.,” and “I react with incomprehension.”) were used to assess anger reactions. Again, responses were given on a four-point Likert scale ranging from “completely agree” to “completely disagree.” As in previous studies (Angermeyer and Matschinger, 2003; Knesebeck et al., 2018), the items were summed up to build an anger scale (range 0–9; Cronbach’s Alpha 0.71).

### Analyses

Pearson’s chi-square test (items) and analyses of variance (scale) were applied to test differences in stigma toward male vs. female, migrant vs. non-migrant, and high vs. low occupational status persons with fatigue. For the chi-square tests, the items were dichotomized to compare agreement (yes/no) between the subgroups. In terms of the scale (anger), the corresponding items were summed up. Percentages (items), means as well as standard errors (scale) and significances (*p*-values) are reported. Statistical procedures were performed with the statistical program package SPSS 27.

## Results

**Figure 1:**
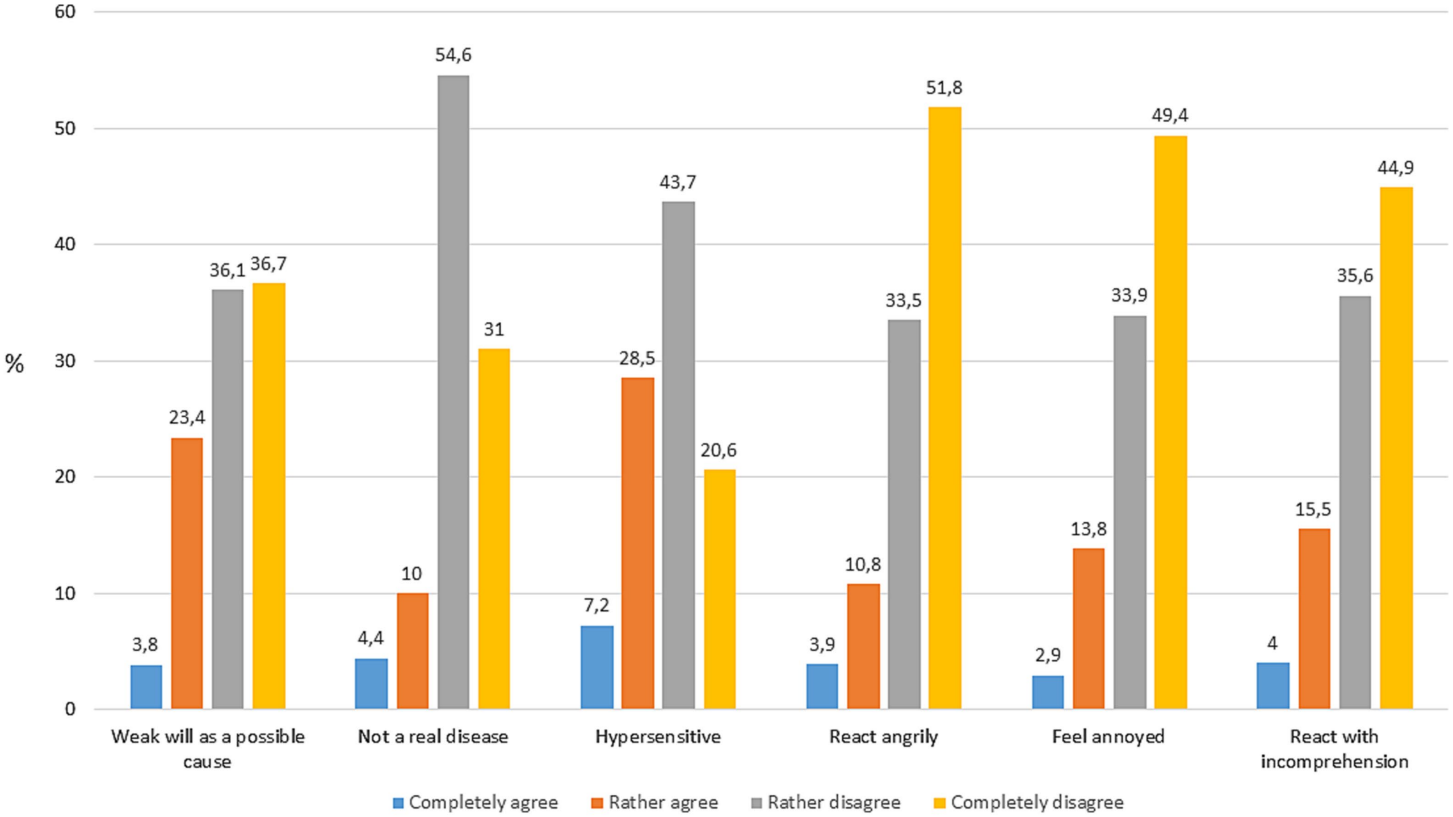
Distribution of the public stigma indicators (*n* = 1,209).

A description of the socio-demographic characteristics of the used sample is shown in Table 2. About 27% of the respondents completely or rather agreed that a possible cause for the symptoms of the person depicted in the vignette is a weak will (Figure 1). About 14% agreed that the person in the vignette does not have a real disease, and more than 1/3 indicated that the person is hypersensitive. In terms of emotional reactions, about 15–20% of the respondents agreed that they react with anger, annoyance or incomprehension in case of a person showing symptoms of fatigue.

**Table 2:** Sample description.

	*n*	%
**Gender**		
Female	634	52.7
Male	570	47.3
**Age groups (in years)**		
18–24	114	9.4
25–39	272	22.5
40–59	379	31.3
60–64	118	9.7
≥65	326	27.0
**Education (in years)**		
≤9	351	30.8
10	374	32.8
≥12	415	36.4
**Household income (per month, net, in euro)**		
<1,500	243	24.9
1,500 – <2,500	289	29.7
2,500 – <3,500	170	17.5
≥3,500	271	27.9
**Migration status**		
Non-migrant	945	78.4
Migrant foreign born	127	10.5
Migrant born in Germany	134	11.1

Table 3 shows that, in case of a male vignette, significantly more respondents agreed that the person is hypersensitive. Also, more respondents reacted with incomprehension in case of the male vignette. However, when the person in the vignette was female, significantly more respondents felt annoyed. In terms of migration background, there were two significant differences in public stigma: Attribution to a weak will and anger reactions were less pronounced when the person with fatigue symptoms was a migrant. With regard to occupational status, significantly more respondents agreed that the person is hypersensitive in case of a lawyer, while more respondents reacted angrily when the depicted person was a cleaner.

**Table 3:** Stigma toward an individual with fatigue depending on social characteristics of the afflicted person in the vignette (sex, migration, occupation).

Stereotypes	Female (*n* = 609)	Male (*n* = 600)	*p* ^b^	Migrant (*n* = 597)	Non-migrant (*n* = 612)	*p* ^b^	Cleaner (*n* = 591)	Lawyer (*n* = 618)	*p* ^b^
Weak will^a^	25.6	28.9	0.214	**23.2**	**31.0**	**0.003**	28.9	25.5	0.201
Not a real disease^a^	15.1	13.6	0.448	15.8	13.2	0.204	13.4	15.3	0.361
Hypersensitive^a^	**31.9**	**39.6**	**0.006**	35.4	35.9	0.865	**32.8**	**38.6**	**0.040**
**Emotional reactions**									
Angrily^a^	15.4	13.9	0.461	13.4	15.9	0.224	**17.6**	**11.8**	**0.004**
Annoyed^a^	**20.9**	**12.2**	**<0.001**	15.9	17.4	0.461	16.5	16.7	0.916
Incomprehension^a^	**16.2**	**22.9**	**0.004**	19.7	19.4	0.878	17.5	21.5	0.083
Anger scale (0–9), mean (standard deviation)	2.16 (2.01)	2.14 (1.92)	0.922	**2.02 (2.04)**	**2.28 (1.89)**	**0.027**	2.14 (2.02)	2.16 (1.91)	0.841

a % of agreement.

b Significance of Chi^2^ tests/analyses of variance (anger scale).Significant differences (*p* < 0.05) are bold.

Differences between the eight combinations of the social characteristics depicted in the fatigue vignettes are shown in Table 4. These differences are significant for all indicators of stigma under study (*p* < 0.05). In terms of weak will, agreement varies between 14.3% (female cleaner with migrant background) and 35.1% (male cleaner with migrant background). 6.3% of the respondents agreed that the person in the vignette does not have a real disease in case of a female cleaner with migrant background, 25.6% agreed for the male counterpart. Variance of agreement was 25.5 to 43.2% for the stereotype ‘hypersensitive’. When the person in the vignette was a male migrant lawyer, 8% reacted angrily, 13.5% less than for the non-migrant counterpart. Variance of those who felt annoyed was from 7.2% (male, non-migrant, lawyer) to 29.6% (female, non-migrant, lawyer). In terms of incomprehension, agreement varied from 10.6 to 27.6% and for the anger sum scale, mean was lowest in case of a male lawyer with a migrant background and highest in case of a female, non-migrant lawyer.

**Table 4:** Stigma toward an individual with fatigue depending on the combination of the social characteristics in the eight vignettes.

Stereotypes	Female, migrant, cleaner (*n* = 146)	Female, migrant, lawyer (*n* = 156)	Female, non-migrant, cleaner (*n* = 151)	Female, non-migrant, lawyer (*n* = 156)	Male, migrant, cleaner (*n* = 142)	Male, migrant, lawyer (*n* = 154)	Male, non-migrant, cleaner (*n* = 152)	Male, non-migrant, lawyer (*n* = 153)	*p* ^b^
Weak will^a^	14.3	20.8	32.6	33.8	35.1	23.2	34.0	23.8	<0.001
Not a real disease^a^	6.3	20.7	16.8	17.1	25.0	12.0	7.4	11.9	<0.001
Hypersensitive^a^	25.5	31.3	28.9	41.2	42.0	43.2	35.8	37.7	0.007
**Emotional reactions**									
Angrily^a^	19.9	12.3	14.7	15.5	14.3	8.0	21.5	11.9	0.028
Annoyed^a^	14.5	20.9	17.9	29.6	18.6	9.2	15.0	7.2	<0.001
Incomprehension^a^	15.9	13.8	10.6	24.5	27.6	22.2	16.6	25.8	<0.001
Anger scale (0–9), mean (standard deviation)	1.92 (2.07)	2.06 (2.12)	2.04 (1.86)	2.61 (1.93)	2.22 (2.11)	1.90 (1.84)	2.38 (2.05)	2.09 (1.66)	0.030

a % of agreement.

b Significance of Chi^2^ tests/analyses of variance (anger scale).

## Discussion

In this study, the magnitude of public stigma in terms of stereotypes and anger toward individuals with fatigue was explored by using a vignette based population survey conducted in Germany. Of the stereotypes under study, “hypersensitive” was most frequently endorsed by the respondents (35.7% completely or rather agreed), followed by “weak will” (27.2% agreement). About 15–20% of the respondents agreed that they react with anger, annoyance or incomprehension in case of a person showing symptoms of fatigue. We additionally analysed differences in stigma depending on the social characteristics of the affected person (sex, occupation, and migration). Accordingly, autochthonous individuals with fatigue were more often stigmatized than those with a Turkish migrant background, while differences according to sex and occupational status (low: cleaner vs. high: lawyer) were inconsistent. A more detailed analysis regarding the combinations of the social characteristics revealed considerable differences between the eight subgroups. In fact, there were differences of about 20% in the endorsement of stereotypes according to the combined social characteristics although the fatigue symptoms described in the vignette were the same. In two social groups public stigma was particularly pronounced: (1) male persons with a low occupational status and a migration background; (2) female persons with a high occupational status and without a migration status. In contrast, women with a low occupational status and a migration background were less stigmatized.

This is one of the first studies analysing public stigma toward people affected by fatigue. There was one previous study with a similar design on public stigma related to SSD (Knesebeck et al., 2018, 2020). Levels of anger reactions as well as endorsement of a weak will were similar to those found in the present study. Our findings can also be compared with previous studies on public stigma toward people with (mental) disorders. In this regard, occurrence of anger reactions was similar in a study on public depression stigma (Knesebeck et al., 2017), while there were lower levels of anger in cases of bulimia nervosa and anorexia nervosa (Makowski et al., 2015). Attribution to a weak will, however, was more pronounced among persons with these eating disorders than in the present study on fatigue. In part, these differences between the results can be explained by varying sample characteristics of the studies. On the other hand, it is known that the magnitude of public stigma varies for different conditions (Pecosolido and Martin, 2015; Knesebeck et al., 2018).

In recent years, it has been claimed that stigma research should pay more attention to multiple social identities and their interaction to influence health-related stigma (Turan et al., 2019). Accordingly, individuals can belong to more than one stigmatized group (e.g., someone who suffers from fatigue and has a deprived social status) and, thus, can be exposed to intersectional or multiple stigma. In this regard, differences according to sex and occupational status in our analyses were inconsistent, while stigmatizing beliefs were less pronounced when the person with fatigue symptoms was a migrant. These findings do not support the hypothesis of multiple stigma which is in line with a previous study focussing on depression stigma (Knesebeck et al., 2017). However, other studies found evidence for multiple stigma. For example, in a study by Makowski et al. (2019), public obesity stigma was more pronounced when the afflicted person has a low socio-economic status. Studies exploring whether respondents showed lower levels of stigma when confronted with a vignette depicting an afflicted person with similar social characteristics like themselves revealed inconsistent results (Makowski et al., 2015; Makowski and Knesebeck, 2017). The role of responders’ social background for stigmatizing attitudes toward affected persons with varying social characteristics should be considered in future studies.

To meet the complex nature of intersectional stigma, we additionally applied an intercategorical approach (Turan et al., 2019) by analysing combinations of the three social characteristics. In doing so, large stigma differences between the eight subgroups were found. In terms of the hypothesis of multiple stigma, inconsistent findings emerged. On the one hand, women with a low occupational status and a migration background were less stigmatized, which contradicts the hypothesis. On the other hand, among male persons with a low occupational status and a migration background, fatigue stigma was particularly pronounced, supporting the hypothesis, at least with regard to migration and socio-economic status. One possible explanation of these inconsistent findings might be that we combined social characteristics (sex, occupation, and migration) representing different horizontal and vertical dimensions of social inequalities. These dimensions can be associated with different forms of discrimination and thus, may be differently connected with disease related stigma. Our results overall indicate that the magnitude of intersectional stigma appears dependent on the combination of the potentially stigmatized social characteristics.

Several limitations should be considered when interpreting present results. In terms of sample quality, 55% of the selected eligible persons refused to participate or were not available and, thus, selection bias cannot be ruled out. On the other hand, comparison of sociodemographic characteristics in the sample with official statistics did not indicate that distribution of age, gender, and education is different from the general adult population in Germany. Although analyses were based on instruments measuring public stigma that were used in previous studies (McLoed et al., 2007; Alvarez-Galvez and Salvador-Carulla, 2013; Angermeyer et al., 2013; Knesebeck et al., 2018), the number and range of indicators of stereotypes and emotional reactions were limited. While the use of vignettes as a standardizing stimulus can be considered established in stigma research, they have to be short to be included into surveys. This affected the presentation of the fatigue symptoms and of the varied social characteristics. The fatigue vignette was developed with the input of clinicians but had to be limited to the major symptoms. Moreover, description of duration of symptoms in the vignette was not substantiated by giving an exact time period. We used unlabelled vignettes, i.e., the respondents were not informed that the person in the vignette had fatigue. Thus, their answers referred only to the described symptoms in the vignette. To distinguish the social groups according to migration and occupational status, only the first sentence in the vignette was varied. Furthermore, only two groups for each of the social characteristics were compared (lawyer vs. cleaner and Turkish migrant background yes vs. no). This must be considered a simplified way to represent aspects of social inequalities. Moreover, other social characteristics of the affected person (e.g., education, religion) that may be relevant for public fatigue stigma, were not considered in the present study. Finally, we cannot exclude that social desirability may have influenced responses regarding stigmatizing attitudes.

It is known that public stigma imposes a great burden on those affected with manifold negative consequences. Results presented here indicate that individuals suffering from fatigue symptoms are confronted with stereotypes and negative emotional reactions by the public. Magnitude of public stigma varies according to social characteristics of the afflicted person. Men with fatigue symptoms, a low occupational status and a migration background seem to be affected by multiple stigma. Future studies analysing public fatigue stigma should consider applying an intersectional approach to identify groups that are at special risk of being stigmatized. In terms of practical implications, three strategies to reduce stigma have been suggested (Corrigan and Penn, 1999): protest or social activism, education of the public, and contact to those affected. A meta-analysis of studies on effects of these anti-stigma approaches came to the result that depending on the target group, education and contact had positive effects on reducing stigma (Corrigan et al., 2012). Our results suggest to consider social characteristics of the affected persons in respective interventions.

## Data availability statement

The raw data supporting the conclusions of this article will be made available by the authors, without undue reservation.

## Ethics statement

The study design was approved by the Ethics Commission of the Hamburg Medical Chamber (No. 2020-10194-BO-ff). The studies were conducted in accordance with the local legislation and institutional requirements. Written informed consent for participation was not required from the participants or the participants' legal guardians/next of kin because Oral informed consent was given in the beginning of the telephone interview.

## Author contributions

OK designed the study, interpreted the data, and drafted the manuscript. RB conducted the analyses, critically revised the manuscript, and approved the final version. All authors contributed to the article and approved the submitted version.

## Funding

This work was carried out within the framework of Research Unit 5211 (FOR 5211) “Persistent SOMAtic Symptoms ACROSS Diseases: From Risk Factors to Modification (SOMACROSS),” funded by the German Research Foundation (Deutsche Forschungsgemeinschaft, DFG). The DFG grant number for this project (P 06) “Social Inequalities in Aggravating Factors of Somatic Symptom Persistence (SOM.SOC)” is 445297796.

## Conflict of interest

The authors declare that the research was conducted in the absence of any commercial or financial relationships that could be construed as a potential conflict of interest.

## Publisher’s note

All claims expressed in this article are solely those of the authors and do not necessarily represent those of their affiliated organizations, or those of the publisher, the editors and the reviewers. Any product that may be evaluated in this article, or claim that may be made by its manufacturer, is not guaranteed or endorsed by the publisher.

## Appendix

Vignettes (two examples).

Ten years ago, 37-year-old Gülsen E. came to Germany from Turkey and she works as a cleaner.^1^ She has been suffering from total exhaustion for some time now. She feels constantly tired, weak and lacking concentration despite an increased number of breaks. The troubles restrict Ms. E. very much in her everyday life and sometimes she cannot go to work. A few months ago, she had a virus infection. Current examinations have not provided any indication of a threatening illness.

37-year-old Martin E. works as a lawyer (see footnote 1). He has been suffering from total exhaustion for some time now. He feels constantly tired, weak and lacking concentration despite an increased number of breaks. The troubles restrict Mr. E. very much in his everyday life and sometimes he cannot go to work. A few months ago, he had a virus infection. Current examinations have not provided any indication of a threatening illness.
